# Divergent gene signatures and neutrophil enrichment in lymph nodes of inflammatory arthritis patients

**DOI:** 10.1186/s13075-025-03557-0

**Published:** 2025-04-23

**Authors:** Aoife M. O’Byrne, Janne W. Bolt, Chaja M.J. van Ansenwoude, Martijn van der Heide, Johanna F. Semmelink, Aldo Jongejan, Perry D. Moerland, Mario Maas, Marleen G.H. van de Sande, Lisa G.M. van Baarsen

**Affiliations:** 1 Amsterdam UMC, Department of Rheumatology & Clinical Immunology, University of Amsterdam, Amsterdam, The Netherlands; 2 Amsterdam UMC, Department of Experimental Immunology, University of Amsterdam, Amsterdam, The Netherlands; 3 Amsterdam Institute for Immunology & Infectious Diseases, Amsterdam, The Netherlands; 4 Amsterdam Rheumatology and Immunology Center (ARC), Amsterdam, The Netherlands; 5 Amsterdam Public Health, Methodology, Amsterdam, The Netherlands; 6 Amsterdam UMC, University of Amsterdam, Epidemiology and Data Science, Amsterdam, Netherlands; 7 Amsterdam UMC, Department of Radiology, University of Amsterdam, Amsterdam, The Netherlands

**Keywords:** Inflammatory arthritis, Neutrophils, Lymph nodes, Pathogenesis

## Abstract

**Background:**

Lymph node (LN) studies in anti-cyclic citrullinated protein antibodies (ACPA) positive rheumatoid arthritis (RA) patients have revealed notable alterations in adaptive immune cell populations. However, it remains unclear whether similar changes occur in seronegative inflammatory arthritis, such as psoriatic arthritis (PsA) or ACPA-negative RA. This study investigates molecular and cellular alterations in LN biopsies from ACPA-positive RA patients, ACPA-negative inflammatory arthritis (IA) patients, and healthy controls (HCs).

**Methods:**

Ultrasound-guided LN biopsies were collected from 25 HCs, 14 ACPA positive RA patients and 45 ACPA negative IA patients (including various IA subtypes). Whole LN tissue biopsies were analyzed by transcriptome analyses, quantitative PCR and immunohistochemistry.

**Results:**

Distinct LN gene expression profiles were identified in ACPA-positive RA and ACPA-negative IA patients compared to HCs. ACPA-positive RA patients exhibited upregulation of genes associated with adaptive immunity, while ACPA-negative IA patients showed higher expression of genes related to innate immune cell function. Subsequent qPCR analysis confirmed increased mRNA expression of Cathepsin G, a serine protease highly expressed by neutrophils, in ACPA negative IA patients. Immunohistochemistry demonstrated significantly elevated CD15 + neutrophil presence in LNs from IA patients compared to HCs, irrespective of ACPA status and diagnosis (RA or PsA).

**Conclusions:**

This study provides novel insights into the immune landscape of lymph nodes in inflammatory arthritis, emphasizing an unexpected role for neutrophils in IA patients. Future research should explore the functional implications of neutrophils within these uninfected lymph nodes to better understand their contribution to the pathogenesis of inflammatory arthritis.

**Supplementary Information:**

The online version contains supplementary material available at https://doi.org/10.1186/s13075-025-03557-0.

## Background

Inflammatory arthritis (IA) encompasses a number of immune mediated inflammatory disorders, which manifest in the synovial joints where immune cells infiltrate and drive a chronic inflammatory environment. If left untreated this leads to pannus formation and bone destruction significantly affecting patients’ quality of life. IA can be further characterized based on symptoms, the main joints involved and the presence of autoantibodies. Rheumatoid arthritis (RA) is a subtype of IA, which preferentially affects peripheral small joints and in most cases includes the presence of anti-cyclic citrullinated protein antibodies (ACPA) [1, 2]. These autoantibodies can be detected years before diagnosis and indicate a break of immune tolerance that precedes clinical onset [2, 3]. Secondary lymphoid organs such as lymph nodes (LN) maintain peripheral tolerance and prevent autoreactive immune cells from initiating systemic autoimmunity. In the past decade our research group has set up the infrastructure to study LN tissue biopsies of early RA patients and those at risk of developing RA (RA-risk) [4–10]. These studies showed significant alterations in cells involved in adaptive immunity in LN biopsies of RA patients and RA-risk individuals compared to healthy controls (HCs), which did not differ based on ACPA positivity or arthritis location [11, 12]. Initial cellular phenotyping revealed that RA and (a subset of) RA-risk individuals have increased frequencies of CXCR3 + CCR6 − CCR4 − Th1 cells [3], ILC1 (c-Kit-NKp44 − ILCs) [12], memory CD8 + T cells [11], CD69 + CD8 + T cells, follicular helper T cells [13], and CD19 + B cells compared to HCs [14]. Additionally, we have shown that the frequency of myeloid and plasmacytoid dendritic cells were increased in LN biopsies of RA patients compared to HCs and RA-risk individuals [15]. This data highlights the additive value of LN tissue analysis in elucidating the immune responses involved in disease pathogenesis. As it has been suggested that the pathogenesis of ACPA negative RA differs from ACPA positive RA [16–18] and that immune cell involvement is linked to autoantibody status [19, 20] we aimed to investigate whether the molecular and cellular landscape in lymphoid organs differs between ACPA positive RA patients and ACPA negative IA patients. Accordingly, this study employs gene profiling in whole tissue biopsies of ACPA negative IA patients compared to ACPA positive RA patients and HCs in an effort to highlight factors or cell populations that may contribute to altered disease pathogenesis between these different forms of IA.

## Methods

### Study cohort and LN biopsy sampling

Patients were recruited at our outpatient-clinic and included in a single-center cross-sectional study. We included 14 ACPA positive RA patients, 45 ACPA negative IA patients and 25 HCs who underwent ultrasound-guided inguinal LN core needle biopsy sampling as previously described [9]. ACPA negative IA patients consisted of patients with a diagnosis following disease classification criteria of RA [21] (23 patients), PsA [22] (12 patients), systemic lupus erythematosus [23] (1 patient), or a clinical diagnosis of crystal arthropathy (2 patients) or undifferentiated arthritis (UA) (7 patients). Patients did not take biological disease modifying anti-rheumatic drugs (bDMARDs) in the 3 months prior to LN sampling however co-medication including NSAIDs, corticosteroids, and conventional DMARDs were permitted. HCs did not have rheumatic disease and were negative for ACPA and rheumatoid factor (RF). A demographics table for all individuals included in this study can be found in Table 1. In the supplementary material we provide demographics tables stratified by analysis method. All patients provided written informed consent prior to enrolment and the study protocol was approved by the Ethics Committee of the Amsterdam Medical Center, Amsterdam, the Netherlands.

**Table 1:** Patient demographics for individuals of study cohort

Total cohort							
Clinical parameter	HC *n* = 25	ACPA- RA *n* = 23	PsA *n* = 12	UA *n* = 7	CA *n* = 2	SLE *n* = 1	ACPA + RA *n* = 14
Age, years (median (IQR))	33 (23-54.5)	62 (48–69)	46 (32.25–56.50)	49 (26–63)	60 (59–60)	38	56 (40.75-60.0)
Gender (male/female)	9/16	7/16	6/6	2/5	2/0	0/1	6/8
Disease duration, months (median (IQR))	-	3 (1–36)	12 (1–21)	1 (0–1)	1 (1–1)	0	5 (0–21)
DAS-28 (median (IQR))	-	3.78 (2.63–4.75)	4.015	4.06 (2.74–5.10)	2.7 (2.02–3.38)	6.2	4.020 (2.713–4.573)
Tender joint count 68 (median (IQR)	-	3 (1.0–17.0)	6.5 (2.25–17.75)	8 (3–17)	1.5 (1.0–2.0)	4	5.0 (0.75–11.25)
Swollen joint count 68 (median (IQR)	-	2 (0.0–5.0)	2.5 (1.25–5.75)	4 (2–5)	1 (1.0–1.0)	4	5.0 (1.5–9.25)
IgM RF positivity (yes/no)	-	7/16	1/11	2/5	0/2	0/1	11/3
CRP (mg/dl) (median (IQR))	0.5 (0.30–2.25)	3.55 (1.05–7.35)	4.95 (1.55–14.48)	2.9 (1.7–3.8)	1.9 (1.6–2.2)	9.0	4.6 (1.50–17.50)
sDMARD use (yes/no)	0/7	7/16	4/12	0/7	0/2	0/1	4/10
Prednisone use (yes/no)	0/7	1/22	0/7	0/7	0/2	0/1	0/14

Categorical variables: (yes/no). Continuous variables (data not normally distributed): median (IQR). HC; healthy controls, UA; undifferentiated arthritis, CA; crystal arthropathy, PsA; psoriatic arthritis, SLE; systemic lupus erythematosus, ACPA; anti-cyclic citrullinated protein antibodies, RA; rheumatoid arthritis, DAS; disease activity score, RF; rheumatoid factor, ESR; erythrocyte sedimentation rate, CRP; C-reactive protein, sDMARD; synthetic disease modifying anti-rheumatic drug

### Genome-wide transcriptome analyses of whole LN tissue biopsies

Fresh biopsies were immediately snap frozen and stored in liquid nitrogen until processing. Total RNA was extracted from whole LN tissue biopsies using the AllPrep DNA/RNA Mini kit from Qiagen (Venlo, the Netherlands). First, LN tissue was quickly homogenized on ice in RLT-plus buffer containing 1% β-mercaptoethanol and 0.05% Qiagen DX reagent using an IKA T10 basic homogenizer (S 10 N 5-G probe; 3,304,000, Cole-Parmer, USA). The homogenized LN tissue suspension was transferred to a clean RNase-free tube and centrifuged to remove cell debris. Using the cleared cell lysate further RNA extraction was performed according to the manufacturer’s instructions including an on-column DNase digestion using the RNase-Free DNase Set (Qiagen). RNA purity and quantity was measured using the Nanodrop (Nanodrop Technologies, Wilmington, USA) with ND1000 V3.8.1 software (ND1000, Isogen Life Science, Utrecht, the Netherlands). Isolated RNA was stored at − 80° Celsius until further use.

Subsequent quality control, RNA labeling, hybridization and data extraction were outsourced at ServiceXS B.V. (Leiden, The Netherlands). The RNA quality and integrity was determined using Lab-on‐Chip analysis on the Agilent 2100 Bioanalyzer (Agilent Technologies, Inc., Santa Clara, CA, U.S.A.) and the Shimadzu MultiNA RNA analysis chips (Shimadzu Corporation, Kyoto, Japan). Biotinylated cRNA was prepared using the Illumina TotalPrep RNA Amplification Kit (Ambion, Inc., Austin, TX, U.S.A.) according to the manufacturer’s specifications with an input of 200 ng total RNA. Per sample, 750 ng of the obtained biotinylated cRNA samples was hybridized onto the Illumina HumanHT‐12 v4 (Illumina, Inc., San Diego, CA, U.S.A.). Each BeadChip contained twelve arrays. Samples were assigned to the different BeadChips, such that the disease categories were equally divided over the BeadChips. Hybridization and washing were performed according to the Illumina Manual “Direct Hybridization Assay Guide”. Scanning was performed on the Illumina iScan (Illumina, Inc., San Diego, CA, U.S.A.). Image analysis and extraction of raw expression data was performed with Illumina GenomeStudio v2011.1 Gene Expression software with default settings (no background subtraction and no normalization).

Analyses were carried out with Bioconductor (version 3.1) packages using the statistical software package R (version 3.3.1). Raw data normalization was performed on the Illumina sample and control probe profiles by normexp-by-control background correction, quantile normalization, and log2 transformation using the limma package (version 3.28.21). The arrayQualityMetrics package (version 3.28.2) was used to assess that the microarray data was of good quality. Probes with a detection P-value > 0.05 (non-expressed) on all arrays (3,478 of 47,323 probes) were filtered out. Differential expression between the experimental conditions was assessed with a moderated t-test using the linear model framework from the limma package including the consensus within-individual correlation (function ‘duplicateCorrelation’), correcting for an observed batch effect between the first four and the last four BeadChips, age (categorized as young (< 39 years), old (> 53 years), or intermediate) and sex of the subjects. Probes were reannotated using the IlluminaHumanv4.db package (version 1.26.0). Gene set enrichment analysis was performed using CAMERA [24] with a value of 0.01 for the inter-gene correlation, using selected genesets collections (Hallmark collection and the BioCarta, KEGG and Reactome subsets of the C2 collection) from the Molecular Signature Database (MSigDB, version 6.0). P-values were calculated using a two-sided directional test (direction of change, ‘up’ or ‘down’) and corrected for multiple testing using the Benjamini-Hochberg false discovery rate.

### Quantitative PCR

cDNA synthesis (samples in Supplementary Table 2) was performed using Revert Aid H Minus First Strand cDNA synthesis kit (Thermo Fisher Scientific, Waltham, Massachusetts, USA) according to manufacturer’s instructions. qPCR analysis was performed using the TaqMan Universal PCR Master Mix with Taqman assays for CTSG (Hs00175195) and TPSAB1 (Hs07291744) (all Applied Biosystems, Life Technologies, Zwijndrecht, The Netherlands). 18 S RNA (Hs99999901_s1) was used as reference gene and all runs included an arbitrary calibrator sample to correct for inter plate variations. The input consisted of 500 ng cDNA with a total reaction volume of 10 µL. Experiments were run on QuantStudio 3 Real-Time PCR System and analysed using QuantStudio v.1.4.3. (both from Applied Biosystems, Life Technologies, Zwijndrecht, The Netherlands) following the manufacturer’s instructions. The relative gene expression was determined using the delta-delta CT method.

### Immunohistochemistry

Core needle biopsies were snap frozen in Tissue-Tek OCT (Sakura) after which 5μm thick sections were cut using a CM1950 cryostat (Leica Microsystems). LN tissue sections were thawed at room temperature followed by fixation with acetone ACS absolute (1000142511, Merck) pure. Sections were washed with phosphate-buffered saline (PBS) and incubated with endogenous peroxidase block (0.1% NaN3 in PBS + 0.35% H_2_O_2_) to reduce nonspecific background staining. The sections were then washed with PBS and incubated overnight at 4°C with primary antibody in PBS with 1% bovine serum albumin (BSA).The following primary antibodies were used: CD15 (IgM, clone HI98, eBioscience, 0.42 µg/ml) or isotype matched antibody as negative control. After washing with PBS the sections were incubated at room temperature with corresponding secondary antibody: streptavidin/HRP labelled donkey IgM, (6.25 µg/ml, DAKO) in PBS containing 10% human serum and 1% BSA (1%). Sections were then washed with PBS and incubated with 3-amino-9-ethylcarbazole (AEC) substrate kit for peroxidase activity (Vector Laboratories) according to manufacturer’s protocol. Sections were washed with demineralised water and counterstained with haematoxylin (Merck). Next, sections were washed with cold tap water and air dried before coverslips were mounted with Kaiser’s glycerine. Imaging was performed on a DMLB + DFC420 light microscope (Leica Microsystems). Stained tissue sections were evaluated by semi-quantitative scoring by three blinded independent observers using a 4-point scale with 0 indicating no presence of staining.

### Statistical analysis

Statistical testing was performed using GraphPad Prism v.8.0.1. Numerical differences between study groups were analysed using Mann-Whitney U or the Kruskal-Wallis test with Dunn’s post hoc test, where appropriate. Correlation coefficients were examined using the Spearman’s correlation. P-values < 0.05 were considered statistically significant.

## Results

### Differential gene signatures in LNs of patients with ACPA negative inflammatory arthritis and ACPA positive RA patients compared with healthy controls

To identify molecular changes in the LN of IA patients we performed an exploratory genome-wide transcriptional profiling study using whole tissue biopsies. We stratified IA patients based on their ACPA status and compared distinct gene expression profiles in ACPA negative IA patients and ACPA positive RA to HCs independently.

**Fig. 1:**
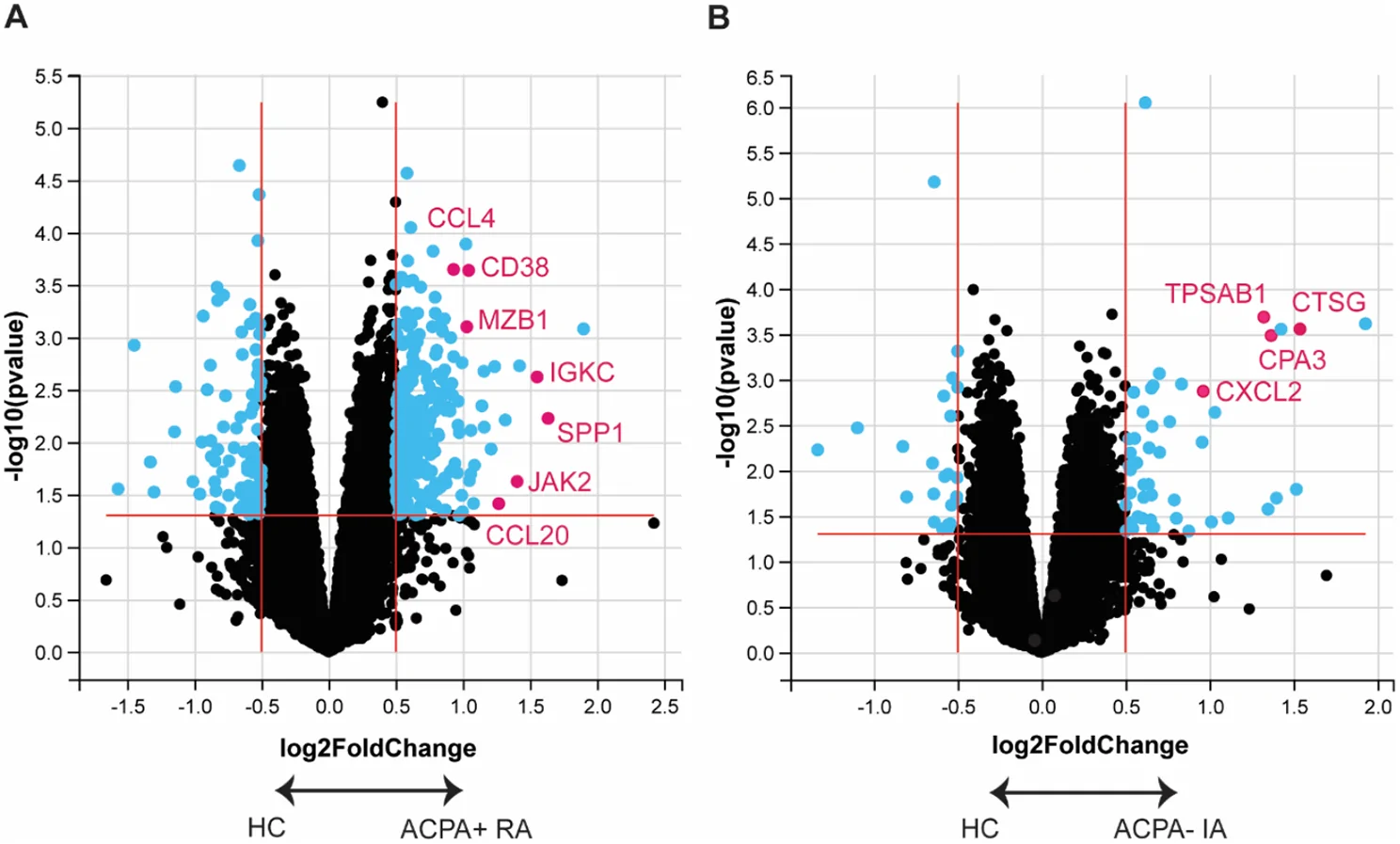
Gene expression profiling of LN biopsies from IA patients and healthy individuals Volcano plots depicting differentially expressed genes of ACPA + RA (**A**) and ACPA- IA (**B**) compared to HCs. Cut of values of *p* = 0.05 and log2-foldchange = 0.5 are represented by red lines. Healthy control (HC), anti-cyclic citrullinated protein antibodies (ACPA), rheumatoid arthritis (RA), inflammatory arthritis (IA)

Differential expression analysis of ACPA positive RA compared to HCs resulted in 336 differentially expressed (absolute log2-foldchange > 0.5 and p-value < 0.05) genes. This distinct gene expression profile involved genes linked to adaptive immune responses (Fig. 1A, Supplementary Table 5) including CD38, a cyclic ADP ribose hydrolase glycoprotein, found on the surface of activated B and T cells. Genes associated with lymphocyte function were also differentially expressed such as Immunoglobulin Kappa Constant (IGKC) and Marginal Zone B1 (MZB1) which are involved in immunoglobulin formation, early T cell signalling molecules, Secreted Phosphoprotein 1 (SPP1) and Janus kinase 2 (JAK2) as well as T cell chemokine ligands CCL20 and CCL4. These observations within ACPA positive RA LNs aligned with our earlier phenotyping data^4,12,15^ which point towards an overall increased adaptive immune cell gene signature in ACPA positive RA compared to HCs. Geneset enrichment analysis (GSEA), focusing on HALLMARK, BIOCARTA, KEGG and REACTOME pathways revealed increased expression of genes linked to cell cycle and DNA replication reflecting a more activated LN environment in ACPA positive RA patients compared to HCs (Table 2).

Differential expression analysis of ACPA negative IA patients compared to HCs resulted in 69 differentially (absolute log2-foldchange > 0.5 and p-value < 0.05) expressed genes. These genes highlighted differences in innate immune responses (Fig. 1B, Supplementary Table 6). Expression of genes encoding, enzymes, tryptase alpha beta 1 (TPSAB1) and carboxypeptidase A3 (CPA3), involved in mast cell degranulation were increased in ACPA negative IA patients compared to HCs. Additionally, Cathepsin G (CTSG), a neutrophil specific serine protease, and CXCL2, a major chemoattractant for neutrophils, were also differentially expressed. Differential expression analysis was also performed on ACPA negative RA and PsA patients compared to HCs (Supplementary Fig. 1 /Table 7) to ascertain whether heterogeneity within the ACPA- IA group was masking significant DEGs. However this analysis did not highlight additional DEGs and resulted in a reduced power likely due to the reduction in the sample size. Subsequent GSEA of ACPA negative IA patients compared to HCs showed decreased expression of gene sets associated with mitochondrial function and the Krebs cycle. This GSEA analysis (Table 3) also highlighted an increased expression of genes related to lysosomal activity, including a number of lysosomal genes expressed in neutrophil granules. Additional to CTSG, Cathepsin C, Cathepsin L1 and cystinosin were all upregulated within the KEGG lysosomal pathway and have been associated with lysosomal activity in neutrophils. We also observed an upregulation of a latent tuberculosis infection pathway which included a number of genes involved in microbial defence mechanisms in neutrophils. Neutrophil cytosolic factor components (NCF2, NCF4) and cytochrome B components (CYBA. CYBB) associated with phagocytic activity, lactoferin associated with secondary neutrophil granules and cathelicidin antimicrobial peptide were all upregulated within this pathway. Accordingly, this GSEA demonstrates the enrichment of a number of pathways pointing towards the presence of neutrophils within the LN of ACPA negative IA patients.

These distinctive differences in gene expression profiles observed in uninfected LNs in the absence of ACPA fuelled our interest to further explore this innate immune cell signature. Accordingly, we set out to investigate the presence of mast cells and neutrophils in the LN tissue biopsies.

**Fig. 2:**
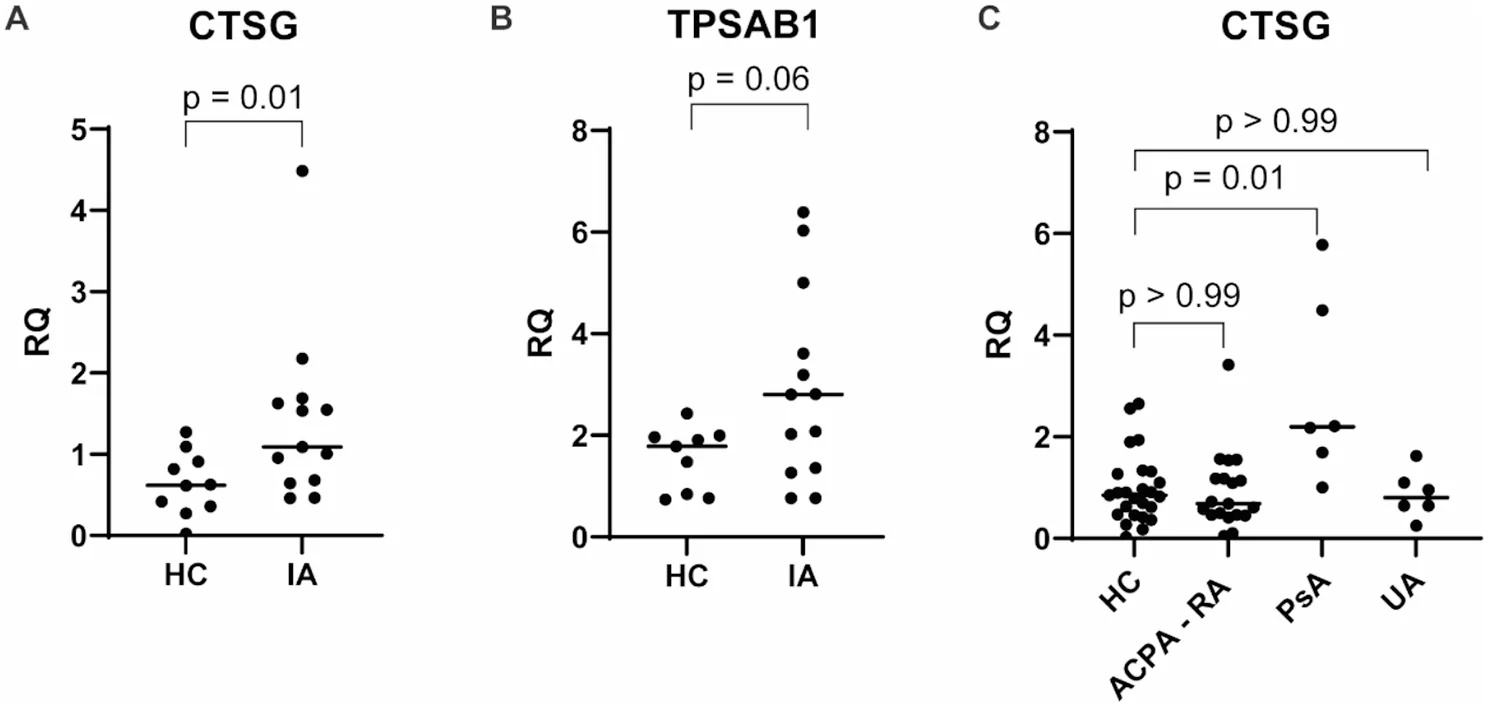
Increased CTSG mRNA levels in LN biopsies of PsA patients Graphs confirming increased mRNA expression levels by qPCR of CTSG (**A**) and TPSAB1 (**B**) in HC (*n* = 10) and ACPA- IA (*n* = 13) samples from the gene expression profiling cohort. CTSG mRNA expression levels (**C**) in HC (*n* = 25), ACPA– RA (*n* = 19), PsA (*n* = 6) and ACPA– UA (*n* = 6) in the validation cohort. All graphs represent median with interquartile range with Mann-Whitney U tests (**A-B**) and Kruskal Wallis test followed by Dunn’s post hoc test (**C**) to determine statistical significance. Cathepsin G (CTSG), relative quantification (RQ), healthy control (HC), inflammatory arthritis (IA), tryptase alpha/beta 1 (TPSAB1), anti-cyclic citrullinated protein antibodies (ACPA), rheumatoid arthritis (RA), psoriatic arthritis (PsA) and undifferentiated arthritis (UA)

**Fig. 3:**
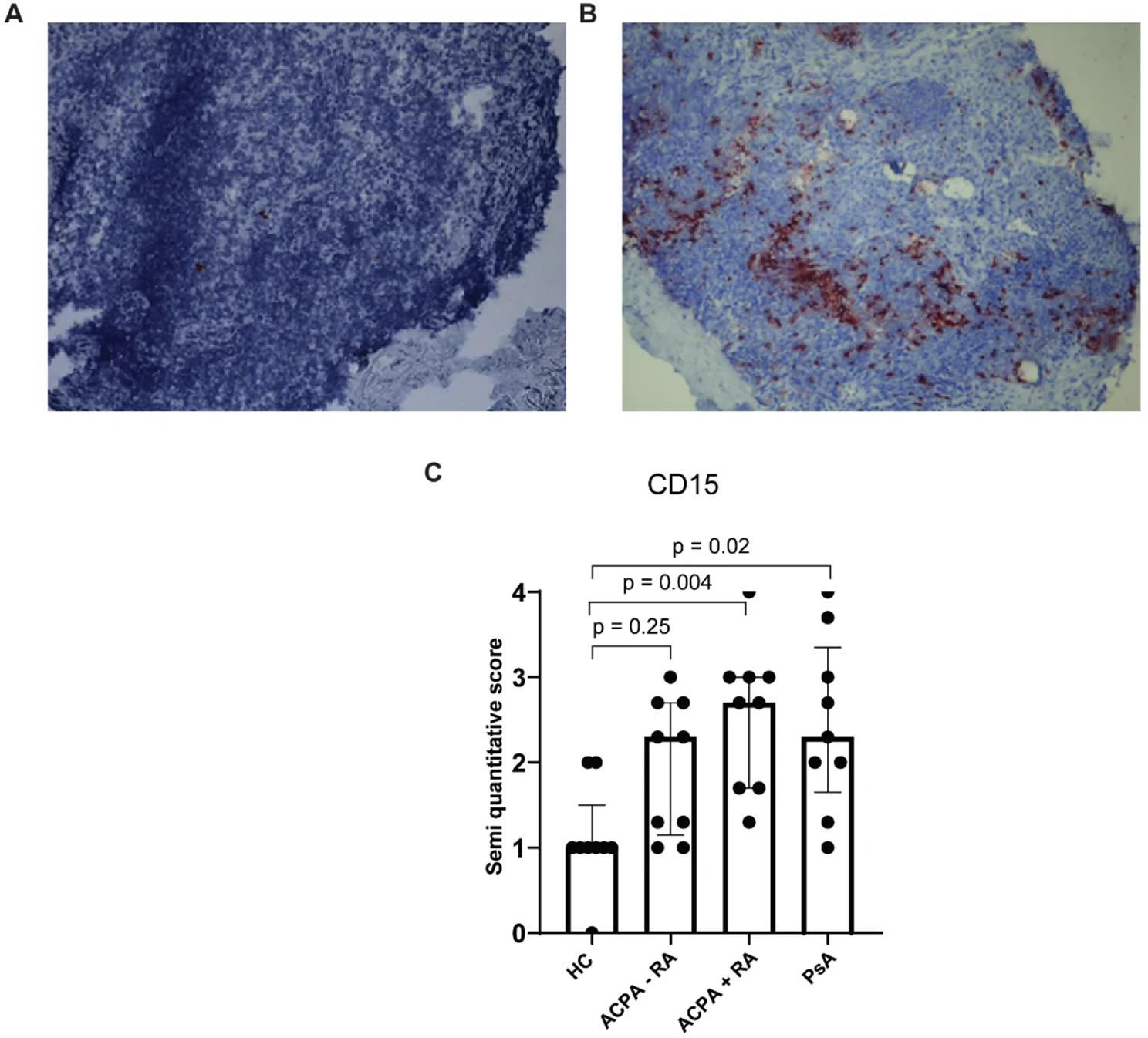
Elevated presence of neutrophils in LN biopsies of patients with inflammatory arthritis. Representative microscopy images showing CD15 staining (dark red) in a LN biopsy section from a HC (**A**) and PsA (**B**). Semi-quantitative score for CD15 abundance (**C**) detected by immunohistochemistry staining in LN tissue sections of HC (*n* = 9), ACPA– RA (*n* = 9), ACPA + RA (*n* = 9) and PsA (*n* = 9). Microscopy images were taken at a 200x magnification. The graph shows median with interquartile range, Kruskal-Wallis test and Dunn’s post hoc test performed to determine statistical significance. Healthy control (HC), anti-cyclic citrullinated protein antibodies (ACPA), rheumatoid arthritis (RA) and psoriatic arthritis (PsA)

**Table 2:** Top 25 differentially expressed genesets in ACPA + RA vs. HCs

GeneSet	Number of Genes in GeneSet	Direction	*P* Value	False Discovery Rate
H_HALLMARK_E2F_TARGETS	190	Up	< 0.0001	< 0.0001
H_HALLMARK_G2M_CHECKPOINT	194	Up	< 0.0001	< 0.0001
C2_REACTOME_DNA_STRAND_ELONGATION	30	Up	< 0.0001	< 0.0001
C2_REACTOME_G2_M_CHECKPOINTS	41	Up	< 0.0001	0.0001
C2_REACTOME_G1_S_SPECIFIC_TRANSCRIPTION	16	Up	< 0.0001	0.0001
C2_REACTOME_DNA_REPLICATION	177	Up	< 0.0001	0.0001
C2_REACTOME_G1_S_TRANSITION	103	Up	< 0.0001	0.0003
C2_REACTOME_UNWINDING_OF_DNA	11	Up	< 0.0001	0.0003
C2_REACTOME_MITOTIC_G1_G1_S_PHASES	127	Up	< 0.0001	0.0003
C2_REACTOME_SYNTHESIS_OF_DNA	87	Up	< 0.0001	0.0003
C2_REACTOME_S_PHASE	103	Up	< 0.0001	0.0003
C2_REACTOME_MITOTIC_M_M_G1_PHASES	158	Up	< 0.0001	0.0004
C2_KEGG_DNA_REPLICATION	36	Up	< 0.0001	0.0005
H_HALLMARK_MYC_TARGETS_V1	194	Up	< 0.0001	0.0005
C2_REACTOME_ASPARAGINE_N_LINKED_ GLYCOSYLATION	75	Up	< 0.0001	0.0006
C2_REACTOME_ACTIVATION_OF_ATR_IN_RESPONSE_TO_ REPLICATION_ STRESS	35	Up	< 0.0001	0.0006
H_HALLMARK_MTORC1_SIGNALING	190	Up	< 0.0001	0.0006
C2_REACTOME_E2F_MEDIATED_REGULATION_OF_DNA_REPLICATION	32	Up	< 0.0001	0.0009
C2_KEGG_N_GLYCAN_BIOSYNTHESIS	43	Up	< 0.0001	0.0009
C2_REACTOME_CELL_CYCLE_CHECKPOINTS	107	Up	< 0.0001	0.0009
C2_REACTOME_CELL_CYCLE_MITOTIC	294	Up	< 0.0001	0.0011
C2_REACTOME_M_G1_TRANSITION	76	Up	< 0.0001	0.0011
C2_REACTOME_ACTIVATION_OF_THE_PRE_REPLICATIVE_COMPLEX	29	Up	< 0.0001	0.0027
C2_REACTOME_CELL_CYCLE	373	Up	< 0.0001	0.0028
C2_REACTOME_REPAIR_SYNTHESIS_FOR_GAP_FILLING_BY_ DNA_POL_IN_TC_NER	14	Up	< 0.0001	0.0034

Table outlining the top 25 differentially expressed genesets in ACPA + rheumatoid arthritis compared to healthy controls following geneset enrichment analysis (GSEA), focusing on HALLMARK, BIOCARTA, KEGG and REACTOME genesets

**Table 3:** Top 25 differentially expressed genesets in ACPA- IA vs. HCs

GeneSet	Number of Genes in GeneSet	Direction	*P* Value	False Discovery Rate
C2_REACTOME_RESPIRATORY_ELECTRON_TRANSPORT	61	Down	< 0.0001	< 0.0001
C2_KEGG_LYSOSOME	110	Up	< 0.0001	< 0.0001
C2_REACTOME_RESPIRATORY_ELECTRON_TRANSPORT_ATP SYNTHESIS_BY CHEMIOSMOTIC_COUPLING_AND_HEAT_PRODUCTION_BY_ UNCOUPLING_PROTEINS_	77	Down	< 0.0001	< 0.0001
C2_REACTOME_FORMATION_OF_THE_TERNARY_COMPLEX_AND_SUBSEQUENTLY THE_43S_COMPLEX	47	Down	< 0.0001	0.0004
C2_REACTOME_TCA_CYCLE_AND_RESPIRATORY_ELECTRON_TRANSPORT	111	Down	< 0.0001	0.0015
C2_REACTOME_INFLUENZA_VIRAL_RNA_TRANSCRIPTION_ AND_REPLICATION	97	Down	< 0.0001	0.0020
C2_REACTOME_3_UTR_MEDIATED_TRANSLATIONAL_ REGULATION	104	Down	< 0.0001	0.0021
C2_KEGG_RIBOSOME	87	Down	< 0.0001	0.0025
C2_REACTOME_ACTIVATION_OF_THE_MRNA_UPON_BINDING_OF_THE_CAP_BINDING_COMPLEX_AND_EIFS_AND_ SUBSEQUENT_BINDING_TO_43S	55	Down	< 0.0001	0.0025
C2_REACTOME_PEPTIDE_CHAIN_ELONGATION	85	Down	< 0.0001	0.0028
C2_KEGG_PARKINSONS_DISEASE	103	Down	< 0.0001	0.0033
C2_KEGG_OXIDATIVE_PHOSPHORYLATION	109	Down	< 0.0001	0.0090
C2_REACTOME_ASPARAGINE_N_LINKED_GLYCOSYLATION	75	Up	0.0001	0.0101
H_HALLMARK_OXIDATIVE_PHOSPHORYLATION	191	Down	0.0001	0.0101
C2_REACTOME_INFLUENZA_LIFE_CYCLE	130	Down	0.0002	0.0118
C2_REACTOME_N_GLYCAN_TRIMMING_IN_THE_ER_AND_ CALNEXIN_CALRETICULIN CYCLE	12	Up	0.0003	0.0218
C2_KEGG_HUNTINGTONS_DISEASE	157	Down	0.0006	0.0370
C2_REACTOME_NONSENSE_MEDIATED_DECAY_ENHANCED_ BY_THE_EXON_JUNCTION_COMPLEX	106	Down	0.0006	0.0370
C2_KEGG_INTESTINAL_IMMUNE_NETWORK_FOR_IGA_ PRODUCTION	43	Up	0.0015	0.0878
C2_REACTOME_LATENT_INFECTION_OF_HOMO_SAPIENS_ WITH_MYCOBACTERIUM_TUBERCULOSIS	28	Up	0.0016	0.0878
C2_REACTOME_METABOLISM_OF_MRNA	208	Down	0.0019	0.0900
C2_REACTOME_TRIGLYCERIDE_BIOSYNTHESIS	38	Down	0.0019	0.0900
C2_KEGG_LEISHMANIA_INFECTION	60	Up	0.0019	0.0900
C2_KEGG_N_GLYCAN_BIOSYNTHESIS	43	Up	0.0019	0.0900
C2_KEGG_ALZHEIMERS_DISEASE	147	Down	0.0022	0.1010

Table outlining the top 25 differentially expressed genesets in ACPA- inflammatory arthritis compared to healthy controls following geneset enrichment analysis (GSEA), focusing on HALLMARK, BIOCARTA, KEGG and REACTOME genesets

### Increased cathepsin G mRNA expression in lymph nodes of ACPA negative PsA patients

We next aimed to validate the upregulation of CTSG and TPSAB1 by qPCR (Fig. 2A-B). CTSG mRNA expression was significantly increased in LNs of ACPA negative IA patients compared to HC (*p* = 0.01, Fig. 2A). TPSAB1 mRNA levels were also higher but did not reach statistical significance (*p* = 0.06, Fig. 2B). Furthermore, qPCR measurements of TPSAB1 showed extremely low mRNA levels in the LN biopsies; reflected by a mean Ct value of 35. In line with this, immunohistochemistry for mast cell tryptase (MCT) showed no presence in almost all LN tissue sections (data not shown), hence the role of mast cells was not explored further.

We next investigated the increased CTSG mRNA expression in a separate larger cohort (Supplementary Table 3) of patients to examine any differences between ACPA negative IA subtypes (ACPA negative RA, PsA and UA). CTSG mRNA expression was significantly increased in PsA LNs compared to HCs (*p* = 0.01, Fig. 2C), while no statistically significant differences were observed between HCs and other ACPA negative IA subtypes.

### Higher frequency of CD15 + neutrophils in LN biopsies of patients with inflammatory arthritis

As CTSG mRNA is highly expressed by neutrophils, we next performed immunohistochemistry on an additional cohort of IA patients (Supplementary Table 4) to study the presence of neutrophils in our LN biopsies. Neutrophils were almost absent in LN tissue sections from HCs (Fig. 3A), in contrast to a clear influx of CD15 + cells observed in LN tissue sections of patients with IA (Fig. 3B). Semi-quantitative analyses showed a high inter-individual variability in neutrophil presence across all patients (Fig. 3C). Elevated levels of CD15 + neutrophils were observed in LNs of IA patients compared to HCs which reached statistical significance for ACPA positive RA and PsA patients (*p* = 0.004 and *p* = 0.02, respectively). Across all patient groups there was a tendency towards a split between high and low neutrophil counts which could not be related to any clinical characteristics, age or sex.

## Discussion

This explorative study is, to our knowledge, the first to examine alterations in LNs of both ACPA positive RA and ACPA negative IA in parallel. Gene expression profiling uncovered key molecular signatures that differed between IA patients based on ACPA status. In ACPA positive RA, we found differentially expressed genes that were predominantly linked to adaptive immunity suggesting an activated LN environment. These results are in line with our previous work exploring LN alterations in RA patients [11–15]. The innate gene signature identified in this study reveals novel insights into the LN environment of patients with IA. The upregulation of gene sets involved in latent microbial infection and lysosomal activity further support an innate immune signature within the LN of ACPA negative IA patients. Altogether, our data highlights an increased influx of neutrophils in LNs of patients with IA irrespective of ACPA status and IA subtype (RA or PsA). The possible explanation for the increased presence of neutrophils in LNs of IA patients is currently unknown as neutrophils are thought to only be present in LNs during infection.

Neutrophils are known to circulate through the blood and lymphatics patrolling for any signs of infection and are the first immune cells present at an injury site. Ample is known about their role at peripheral tissue sites such as the skin where they swarm upon injury and, in the case of an infection, traffic to the draining LN to perpetuate an immune response [25]. Nevertheless knowledge of their function during homeostasis in peripheral tissue and LNs is sparse. Neutrophils were originally thought to be absent or at non-detectable levels in the LN; however, a recent study of healthy human uninfected inguinal LNs show similar levels of neutrophils [26] as we have observed in our healthy controls. Mice studies have suggested that the neutrophils residing in LNs in the absence of an infection [27] are a unique resident population. However, the functional consequence of this or its presence in humans has not been confirmed.

The influx of neutrophils observed in IA patients but not in HCs suggests a possible pathogenic role for these neutrophils; however, whether they are causative or a consequence of chronic inflammation is unclear. In our immunohistochemistry data we observed two distinct patient groups based on neutrophil presence within the three investigated IA subtypes, ACPA positive RA, ACPA negative RA and PsA. However, this was not related to any clinical parameters, or with the presence of active psoriasis lesions in the proximity of the inguinal region of PsA patients. The presence of neutrophils across all patient groups highlights that their presence is not unique to an IA subtype. Neutrophil presence was not related to current or previous treatment indicating that this aberrant expression is not a consequence of therapeutic intervention. It is important to note that although no increased CTSG mRNA expression was observed in the ACPA negative RA group, we did observe increased neutrophil presence at the protein level by immunohistochemistry. The only distinguishing characteristic of the two cohorts is the increased disease duration of the ACPA negative RA compared to ACPA positive RA and PsA (Supplementary Tables 3–4). Future studies exploring neutrophil presence in cohorts with varying disease durations are required to elucidate whether this neutrophil influx is a specific phenomenon observed in early arthritis.

Neutrophils are reported to be abundantly present in the synovium [8, 28] of IA patients and more specifically in the skin [29] and entheses [30] of PsA patients. The presence of neutrophils in these tissues, coupled with the known role of IL-17 producing T cells in PsA synovium [31] and the observed decrease in neutrophils upon successful treatment [6, 8, 32–34], further emphasizes a possibly pathogenic role for neutrophils in arthritis development. It would be of interest to explore the phenotype of these neutrophils residing in the LN as current evidence in mice and human LN suggests they have increased HLA-DR expression [26] compared to those present in the spleen or blood. Neutrophil trafficking studies in mice [35, 36] and in-vitro human co-culture experiments also suggest that neutrophils may function as early vessels for antigens [37, 38].

This current study is limited by the small sample size of our validation cohort for neutrophil presence. Nonetheless, considering the difficulty in obtaining these unique LN biopsies from active patients and healthy individuals, these findings clearly highlight a neutrophil population not previously identified in the LN. Human neutrophils, especially in tissues, are notoriously difficult to study. Our gene expression profiling approach using whole tissue biopsies allowed us to uncover a neutrophil gene signature which would not have been identified using more advanced single cell based methods due to the low abundance and short lifespan of neutrophils which would have hampered their survival upon tissue processing.

## Conclusions

In conclusion, our study is the first to identify distinct gene signatures in human LN tissue biopsies in ACPA negative IA, highlighting an increased neutrophil presence in LNs during IA in absence of infection. Future studies are required to investigate the potential routes of entry, phenotype and cellular interactions of these LN neutrophils to fully understand their pathogenic role in IA pathogenesis.

## Electronic supplementary material

Below is the link to the electronic supplementary material.

Supplementary Material 1

## Data Availability

The datasets used and/or analysed during the current study are available from the corresponding author on reasonable request.

## Abbreviations

LN: Lymph node
IA: Inflammatory arthritis
RA: Rheumatoid arthritis
PsA: Psoriatic arthritis
ACPA: Anti-citrullinated peptide antibodies
HC: Healthy controls
