# Correction: Divergent gene signatures and neutrophil enrichment in lymph nodes of inflammatory arthritis patients

**DOI:** 10.1186/s13075-026-03936-1

**Published:** 2026-10-27

**Authors:** Aoife M. O’Byrne, Janne W. Bolt, Chaja M. J. van Ansenwoude, Martijn van der Heide, Johanna F. Semmelink, Aldo Jongejan, Perry D. Moerland, Mario Maas, Marleen G. H. van de Sande, Lisa G. M. van Baarsen

**Affiliations:** 1 Amsterdam UMC, Department of Rheumatology & Clinical Immunology, University of Amsterdam, Amsterdam, The Netherlands; 2 Amsterdam UMC, Department of Experimental Immunology, University of Amsterdam, Amsterdam, The Netherlands; 3 Amsterdam Institute for Immunology & Infectious Diseases, Amsterdam, The Netherlands; 4 Amsterdam Rheumatology and Immunology Center (ARC), Amsterdam, The Netherlands; 5 Amsterdam Public Health, Methodology, Amsterdam, The Netherlands; 6 Amsterdam UMC, University of Amsterdam, Epidemiology and Data Science, Amsterdam, Netherlands; 7 Amsterdam UMC, Department of Radiology, University of Amsterdam, Amsterdam, The Netherlands

**Correction: Arthritis Res Ther 27, 94 (2025)**

**https://doi.org/10.1186/s13075-025-03557-0**

Following publication of the original article [1], the author’s name Martijn van der Heide was incorrectly written as Martijn van der Heijde.

This has been corrected above and the original article [1] has been updated.
