# Supplementary material for: Divergent gene signatures and neutrophil enrichment in lymph nodes of inflammatory arthritis patients
Source: Arthritis Res Ther. 2025 Apr 23;27:94. doi: 10.1186/s13075-025-03557-0 (PMC12016067; doi:10.1186/s13075-025-03557-0)
Supplement: Supplementary file 1 — Supplementary Material 1 [file 13075_2025_3557_MOESM1_ESM.docx]

**SUPPLEMENTARY MATERIAL**

Supplementary Table 1. Patient demographics for individuals analyzed by microarray.

Supplementary Table 2. Patient demographics for qPCR validation of microarray data.

Supplementary Table 3. Patient demographics for extended cohort qPCR validation.

Supplementary Table 4. Patient demographics for individuals analyzed by immunohistochemistry.

Supplementary Table 5. Top 60 differentially expressed genes of microarray analyses in ACPA positive RA

Supplementary Table 6. Top 60 differential expressed genes of microarray analyses in ACPA negative IA patients

Supplementary Table 7. Top differential expressed genes of microarray analyses in ACPA negative RA and PsA compared to HC

Supplementary Figure 1. Gene expression profiling of LN biopsies from ACPA negative RA and PsA compared to healthy individuals

| **Clinical parameter** | **HC**  **(n = 10)** | **ACPA- IA**  **(n= 16)** | **ACPA+ RA**  **(n = 8)** |
| --- | --- | --- | --- |
| Age, years (median (IQR)) | 35 (28-51) | 54 (37-62) | 59 (47-61) |
| Sex (male/female) | 3/7 | 6/10 | 3/5 |
| Disease duration, months (median (IQR)) | - | 1  (0.25-2.0) | 0.5  (0-1) |
| DAS-28 (median (IQR)) | - | 3.7  (2.8-5.8) | 4.5  (3.1-4.6) |
| Tender joint count 68 (median (IQR) | - | 11.5  (1.3-20.5) | 5.0  (3.3-8.8) |
| Swollen joint count 68 (median (IQR) | - | 4.5  (1.5-6.0) | 8.0  (2.8-10.8) |
| IgM RF positivity (yes/no) | - | 2/14 | 5/3 |
| CRP (mg/dl) (median (IQR)) | 0.7  (0.4-3.7) | 3.3  (1.2-13.1) | 5.2  (1.4-21.2) |
| sDMARD use n (%) | - | - | - |
| Prednisone use n (%) | - | - | - |

**Supplementary Table 1. Patient demographics for individuals analyzed by microarray**

Categorical variables: n (%). Continuous variables (data not normally distributed): median (IQR). HC; healthy controls, IA; inflammatory arthritis, ACPA; anti-cyclic citrullinated protein antibodies, RA; rheumatoid arthritis, DAS; disease activity score, RF; rheumatoid factor, ESR; erythrocyte sedimentation rate, CRP; C-reactive protein, sDMARD; synthetic disease modifying anti-rheumatic drug.

**Supplementary Table 2. Patient demographics for qPCR validation of microarray data**

| **Clinical parameter** | **HC**  **(n = 10)** | **ACPA- IA**  **(n = 13)** |
| --- | --- | --- |
| Age, years (median (IQR)) | 35 (28-51) | 54 (33-63) |
| Sex (male/female) | 3/7 | 4/9 |
| Disease duration, months (median (IQR)) | - | 1  (0.5-2) |
| DAS-28 (median (IQR)) | - | 4.0  (2.8-5.8) |
| Tender joint count 68 (median (IQR) | - | 12.0  (2.0-20.0) |
| Swollen joint count 68 (median (IQR) | - | 5.0  (3.0-8.5) |
| IgM RF positivity (yes/no) | - | 2/11 |
| CRP (mg/dl) (median (IQR)) | 0.7  (0.4-3.7) | 3.3  (1.0-18.7) |
| sDMARD use n (%) | - | - |
| Prednisone use n (%) | - | - |

* All HC and 13 ACPA - IA patients included in this cohort were also part of the cohort of Supplementary Table 1.
Categorical variables: n (%). Continuous variables (data not normally distributed): median (IQR). HC; healthy controls, PsA; psoriatic arthritis, IA; inflammatory arthritis, ACPA; anti-cyclic citrullinated protein antibodies, DAS; disease activity score, RF; rheumatoid factor, ESR; erythrocyte sedimentation rate, CRP; C-reactive protein.

| **Clinical parameter** | **HC**  **(n = 25)** | **ACPA- RA**  **(n = 19)** | **PsA**  **(n = 6)** | **UA**  **(n = 6)** |
| --- | --- | --- | --- | --- |
| Age, years (median (IQR)) | 33 (28-55) | 62 (54-69) | 48 (37-65) | 40 (26-57) |
| Sex (male/female) | 9/16 | 5/14 | 2/4 | 2/4 |
| Disease duration, months (median (IQR)) | - | 3  (1-48) | 1.0  (0.0-4.0) | 0.5  (0-1) |
| DAS-28 (median (IQR)) | - | 3.8  (2.2-4.7) | 4.4  (3.8-5.2) | 3.4  (2.6-5.2) |
| Tender joint count 68 (median (IQR) | - | 2.0  (0.0-13.0) | 13.0  (6.8-19.5) | 6.5  (2.5-16.5) |
| Swollen joint count 68 (median (IQR) | - | 2.0  (0.0-5.0) | 4.0  (2.5-8.5) | 3.5  (1.8-4.3) |
| IgM RF positivity (yes/no) | - | 5/14 | 1/5 | 2/4 |
| CRP (mg/dl) (median (IQR)) | 0.70  (0.35-2.55) | 3.9  (1.0-8.0) | 14.5  (3.1-22.6) | 3.3  (2.0-17.5) |
| sDMARD use n (%) | - | 37 | - | - |
| Prednisone use n (%) | - | 5 | - | - |

**Supplementary Table 3. Patient demographics for extended cohort qPCR validation***

This cohort includes 13 HC and 13 ACPA – RA patients also included in the cohort of Supplementary Table 1. Continuous variables (data not normally distributed): median (IQR). HC; healthy controls, ACPA; anti-cyclic citrullinated protein antibodies, PsA; psoriatic arthritis, IA; inflammatory arthritis, DAS; disease activity score, RF; rheumatoid arthritis, ESR; erythrocyte sedimentation rate, CRP; C-reactive protein.

**Supplementary Table 4. Patient demographics for individuals analyzed by immunohistochemistry***

| **Clinical parameter** | **HC**  **(n = 9)** | **ACPA- RA**  **(n = 9)** | **ACPA+ RA**  **(n = 9)** | **PsA**  **(n = 9)** |
| --- | --- | --- | --- | --- |
| Age, years (median (IQR)) | 47 (36-62) | 54 (43-62) | 47 (39-59) | 40 (30-52) |
| Sex (male/female) | 4/5 | 3/6 | 4/5 | 5/4 |
| Disease duration, months (median (IQR)) | - | 36  (12-150) | 12  (12-78) | 12  (12-78) |
| DAS-28 (median (IQR)) | - | 2.8  (2.2-5.0) | 3.6  (1.7-5.3) | 4.0  (2.8-5.5) |
| Tender joint count 68 (median (IQR) | - | 1.0  (0.0-14.5) | 3.0  (0.0-11.5) | 5.0  (2.0-10.0) |
| Swollen joint count 68 (median (IQR) | - | 3.0  (0.0-5.0) | 4.0  (0.0-7.0) | 2.0  (1.0-3.0) |
| IgM RF positivity (yes/no) | - | 4/9 | 9/0 |  |
| CRP (mg/dl) (median (IQR)) | 0.50  (0.30-2.50) | 5.2  (1.5-8.1) | 1.9  (1.2-9.5) | 5.3  (1.8-14.5) |
| sDMARD use n (%) | - | 67 | 56 | 56 |
| Prednisone use n (%) | - | 11 | 11 | - |

This cohort includes 4 HC, 1 PsA patient, 3 ACPA – RA and 3 ACPA + RA patients that were also included in the cohort of Supplementary Table 1.
Categorical variables: n (%). Continuous variables (data not normally distributed): median (IQR). HC; healthy controls, ACPA; anti-cyclic citrullinated protein antibodies, RA; rheumatoid arthritis, PsA; psoriatic arthritis, DAS; disease activity score, ESR; erythrocyte sedimentation rate, CRP; C-reactive protein, VAS; visual analogue score.

**Supplementary Table 5. Top 60 differentially expressed genes of microarray analyses in ACPA positive RA**

| **Gene** | **Log2FC** | **Average expression** | **P value** | **Adjusted P value** |
| --- | --- | --- | --- | --- |
| DPAGT1 | 0.401108122 | 8.802350699 | 5.69E-06 | 0.24954776 |
| PPIL3 | -0.664337581 | 10.3443433 | 2.28E-05 | 0.395086743 |
| ENTPD1 | 0.583954477 | 7.432724056 | 2.70E-05 | 0.395086743 |
| WDR33 | -0.517019921 | 6.332154749 | 4.34E-05 | 0.448107267 |
| CTSA | 0.499234448 | 8.347529017 | 5.11E-05 | 0.448107267 |
| HHLA3 | 0.61242316 | 5.20724787 | 8.94E-05 | 0.550578675 |
| RMND1 | -0.525881414 | 6.031929512 | 0.000119215 | 0.550578675 |
| TK1 | 1.023330611 | 5.88376305 | 0.000128557 | 0.550578675 |
| CPNE5 | 0.778286901 | 5.44284284 | 0.000150322 | 0.550578675 |
| TTC38 | 0.475925627 | 7.113021331 | 0.000163355 | 0.550578675 |
| PCOTH | 0.313290683 | 4.425940858 | 0.000184312 | 0.550578675 |
| PHF19 | 0.58932568 | 6.233152885 | 0.000186326 | 0.550578675 |
| CCL4 | 0.931113891 | 7.029988026 | 0.000224599 | 0.550578675 |
| CD38 | 1.043741501 | 8.004552378 | 0.000229245 | 0.550578675 |
| BG951912 | -0.399400548 | 4.606085933 | 0.000252206 | 0.550578675 |
| MED8 | 0.469517228 | 6.975346026 | 0.000255334 | 0.550578675 |
| FEN1 | 0.543167783 | 6.066966762 | 0.000266101 | 0.550578675 |
| CDC25A | 0.627293819 | 4.707673195 | 0.000284702 | 0.550578675 |
| CDYL2 | 0.448989446 | 4.915428443 | 0.000285762 | 0.550578675 |
| TXNDC11 | 0.591101244 | 5.43757672 | 0.000291786 | 0.550578675 |
| LELP1 | 0.298403207 | 4.478017443 | 0.00029611 | 0.550578675 |
| PHF5A | 0.503092934 | 6.327850852 | 0.000312275 | 0.550578675 |
| MCM2 | 0.686508982 | 6.537589635 | 0.000330609 | 0.550578675 |
| RAB38 | -0.831041493 | 6.232702754 | 0.00033205 | 0.550578675 |
| SLC46A2 | 0.445586715 | 4.752631515 | 0.000349061 | 0.550578675 |
| MRPL11 | 0.498978986 | 4.850716872 | 0.000350785 | 0.550578675 |
| CDH23 | -0.782305315 | 5.596304023 | 0.000395749 | 0.550578675 |
| SNX5 | 0.793652284 | 6.127904042 | 0.000413806 | 0.550578675 |
| HBBP1 | -0.826727161 | 5.024935283 | 0.000443609 | 0.550578675 |
| MICA | -0.585979771 | 7.011905357 | 0.000486332 | 0.550578675 |
| PCBP4 | -0.290307849 | 4.46916461 | 0.000525979 | 0.550578675 |
| C15orf2 | 0.44064777 | 4.527047779 | 0.000528713 | 0.550578675 |
| SPAG5 | 0.472581936 | 4.674385439 | 0.000530503 | 0.550578675 |
| C20orf30 | 0.329050976 | 10.98458373 | 0.000554883 | 0.550578675 |
| FAM115C | 0.459610091 | 5.35472502 | 0.000571509 | 0.550578675 |
| C16orf59 | 0.458893293 | 4.727602739 | 0.000572531 | 0.550578675 |
| PIM2 | 0.579339783 | 9.797709904 | 0.000575873 | 0.550578675 |
| CKAP2L | 0.79507795 | 5.123165525 | 0.000588107 | 0.550578675 |
| GCNT3 | 0.664515505 | 4.685735327 | 0.000588337 | 0.550578675 |
| XAGE1 | 0.579295197 | 4.904323827 | 0.00059142 | 0.550578675 |
| TMEM178 | -0.338670038 | 4.550377696 | 0.00060758 | 0.550578675 |
| PDE3B | -0.933750226 | 5.875533922 | 0.000625888 | 0.550578675 |
| DOCK5 | 0.306723909 | 4.529180185 | 0.00063211 | 0.550578675 |
| LOC282997 | -0.542362545 | 5.735193826 | 0.000657211 | 0.550578675 |
| CHEK1 | 0.862125986 | 5.19454429 | 0.00066474 | 0.550578675 |
| DNASE2 | 0.314289203 | 9.144775848 | 0.000672793 | 0.550578675 |
| PAXIP1-AS2 | -0.436218552 | 6.887171254 | 0.00067509 | 0.550578675 |
| CENPA | 0.587277544 | 4.815630955 | 0.000723927 | 0.550578675 |
| CLCC1 | 0.616808745 | 5.59287764 | 0.000734307 | 0.550578675 |
| KIAA2013 | 0.297275899 | 8.626901071 | 0.000736287 | 0.550578675 |
| GATS | -0.582543927 | 6.180511777 | 0.000742391 | 0.550578675 |
| BPTF | -0.510058714 | 5.783149484 | 0.000743748 | 0.550578675 |
| C15orf23 | 0.516758706 | 5.585776137 | 0.000746646 | 0.550578675 |
| USP6NL | 0.475452797 | 7.086252266 | 0.000754648 | 0.550578675 |
| TROAP | 0.830429144 | 5.194078987 | 0.000760678 | 0.550578675 |
| SAR1B | 0.47036423 | 6.619478053 | 0.000768005 | 0.550578675 |
| RSG1 | -0.453760446 | 5.109604557 | 0.000769299 | 0.550578675 |
| RAD51AP1 | 0.593748741 | 6.406640992 | 0.000782348 | 0.550578675 |
| CR2 | 0.774074555 | 9.653393637 | 0.000789542 | 0.550578675 |
| MZB1 | 1.02974617 | 9.926996507 | 0.000792511 | 0.550578675 |

**Supplementary Table 6. Top 60 differential expressed genes of microarray analyses in ACPA negative IA patients**

| **Gene** | **Log2FC** | **Average expression** | **P value** | **Adjusted P value** |
| --- | --- | --- | --- | --- |
| TTC12 | 0.617115 | 5.549908976 | 8.96E-07 | 0.039295834 |
| C17orf75 | -0.63969 | 5.039604098 | 6.68E-06 | 0.146544737 |
| RYR2 | -0.40712 | 4.657158386 | 0.000102 | 0.99988429 |
| SLC46A2 | 0.419144 | 4.767073983 | 0.000191 | 0.99988429 |
| TPSAB1 | 1.322363 | 7.481246053 | 0.000205 | 0.99988429 |
| BC071615 | -0.27845 | 4.460912336 | 0.000219 | 0.99988429 |
| CTSG | 1.538284 | 8.757917934 | 0.000278 | 0.99988429 |
| TPSAB1 | 1.425199 | 8.444938 | 0.000279 | 0.99988429 |
| LOC100130673 | -0.20663 | 4.389478388 | 0.000289 | 0.99988429 |
| CPA3 | 1.365936 | 7.97994455 | 0.000327 | 0.99988429 |
| LOC440149 | -0.31484 | 4.59297682 | 0.000365 | 0.99988429 |
| UBQLN4 | 0.226419 | 8.760687738 | 0.000426 | 0.99988429 |
| CEP68 | -0.5001 | 6.060286654 | 0.000485 | 0.99988429 |
| AW590489 | 0.367246 | 5.027517934 | 0.000505 | 0.99988429 |
| BTBD7 | -0.2824 | 4.433014842 | 0.000521 | 0.99988429 |
| NDUFAF4 | 0.270289 | 4.414822111 | 0.000567 | 0.99988429 |
| POTE2 | -0.33999 | 4.801505048 | 0.000578 | 0.99988429 |
| TSHZ1 | -0.31209 | 7.974462021 | 0.0007 | 0.99988429 |
| SIRT3 | -0.25221 | 4.546605961 | 0.000716 | 0.99988429 |
| RNPS1 | -0.26696 | 4.488234397 | 0.00079 | 0.99988429 |
| CTNNBIP1 | -0.32368 | 4.537947565 | 0.000809 | 0.99988429 |
| RPL35 | -0.38345 | 12.87846337 | 0.00085 | 0.99988429 |
| MRPL43 | -0.31278 | 9.030509386 | 0.000852 | 0.99988429 |
| CRTAM | -0.3694 | 4.730209549 | 0.000853 | 0.99988429 |
| ESPNL | 0.700642 | 5.760582537 | 0.000857 | 0.99988429 |
| HMGB1 | -0.31536 | 9.248643358 | 0.000875 | 0.99988429 |
| C21orf89 | -0.21438 | 4.459307586 | 0.000887 | 0.99988429 |
| ALKBH3 | -0.20813 | 7.57190713 | 0.000892 | 0.99988429 |
| DPAGT1 | 0.282634 | 8.828997879 | 0.000898 | 0.99988429 |
| LOC643980 | -0.31479 | 4.598558036 | 0.000933 | 0.99988429 |
| LOC388789 | -0.19949 | 9.73202973 | 0.000959 | 0.99988429 |
| PPM1A | -0.52961 | 5.394582592 | 0.00096 | 0.99988429 |
| POLR2I | -0.23645 | 9.26838507 | 0.000991 | 0.99988429 |
| CTSA | 0.326761 | 8.335263315 | 0.000993 | 0.99988429 |
| OR10S1 | -0.22554 | 4.474507057 | 0.001018 | 0.99988429 |
| COL4A3BP | -0.32538 | 5.267062026 | 0.001039 | 0.99988429 |
| HDC | 0.833063 | 5.57901766 | 0.001121 | 0.99988429 |
| PSORS1C3 | 0.295116 | 4.486480717 | 0.001134 | 0.99988429 |
| KCTD1 | -0.3581 | 4.70509295 | 0.001166 | 0.99988429 |
| C1orf186 | 0.663643 | 5.40679408 | 0.001167 | 0.99988429 |
| SLC25A10 | 0.49685 | 5.132728765 | 0.001174 | 0.99988429 |
| PLAGL2 | -0.29782 | 4.512899114 | 0.001185 | 0.99988429 |
| RPL31 | -0.50041 | 7.6245407 | 0.001216 | 0.99988429 |
| FAM189B | 0.222811 | 8.301743588 | 0.001227 | 0.99988429 |
| SLC37A2 | 0.653105 | 6.972636243 | 0.001243 | 0.99988429 |
| ZNF805 | -0.27206 | 6.492905618 | 0.001249 | 0.99988429 |
| KIAA0196 | -0.24188 | 8.69735659 | 0.001293 | 0.99988429 |
| LOC653333 | 0.335669 | 4.846313728 | 0.001299 | 0.99988429 |
| TMBIM6 | 0.209828 | 11.97019586 | 0.001339 | 0.99988429 |
| PLEC1 | -0.28412 | 4.501226405 | 0.001341 | 0.99988429 |
| CXCL2 | 0.961631 | 7.534227873 | 0.001341 | 0.99988429 |
| UNC93B1 | 0.548462 | 5.548411756 | 0.001379 | 0.99988429 |
| MAPK1 | -0.44178 | 5.456570037 | 0.001386 | 0.99988429 |
| ZBED3 | -0.38313 | 5.182105575 | 0.001405 | 0.99988429 |
| RIOK3 | -0.27068 | 4.390060828 | 0.001408 | 0.99988429 |
| C20orf181 | -0.30773 | 4.620025044 | 0.001502 | 0.99988429 |
| CACNB3 | 0.409226 | 6.46936949 | 0.001512 | 0.99988429 |
| LOC732381 | -0.58222 | 4.628541809 | 0.001514 | 0.99988429 |
| C8orf86 | 0.251655 | 4.55093328 | 0.001728 | 0.99988429 |
| ARMC10 | -0.28023 | 4.547265985 | 0.001781 | 0.99988429 |
| C14orf176 | -0.32018 | 4.635539577 | 0.001839 | 0.99988429 |

| **Gene** | **logFC** | **Average Expression** | **P value** | **Adjusted P value** |
| --- | --- | --- | --- | --- |
| HLA-A29.1 | 2.924637291 | 9.417742976 | 0.017204 | 0.996471214 |
| TMEM107 | 2.481132591 | 7.018503993 | 0.001143 | 0.996471214 |
| SLN | 2.471301055 | 6.15773276 | 6.03E-05 | 0.996471214 |
| CTSG | 1.956633147 | 8.630159606 | 0.004426 | 0.996471214 |
| LOC652155 | 1.950760534 | 5.632304935 | 0.008104 | 0.996471214 |
| RNF126P1 | 1.858158217 | 8.83736813 | 0.128554 | 0.996471214 |
| TPSAB1 | 1.682019178 | 8.268829555 | 0.010254 | 0.996471214 |
| LOC100131845 | 1.648409057 | 7.431609396 | 0.110355 | 0.996471214 |
| CPA3 | 1.619931916 | 7.866215801 | 0.010756 | 0.996471214 |
| KIAA1199 | 1.606743003 | 11.21834731 | 0.000728 | 0.996471214 |
| SLC25A47 | 1.515105085 | 5.63694665 | 0.023174 | 0.996471214 |
| SCGB3A1 | 1.413566141 | 5.515401726 | 0.008923 | 0.996471214 |
| ACTG2 | 1.364142622 | 10.864929 | 0.097076 | 0.996471214 |
| HLA-DRB5 | 1.297374213 | 7.293565029 | 0.558248 | 0.996471214 |
| TPSAB1 | 1.28502564 | 7.378798473 | 0.023197 | 0.996471214 |
| PSORS1C1 | 1.282828608 | 8.443880151 | 0.177491 | 0.996471214 |
| NRSN2 | 1.269436884 | 8.810885777 | 0.060633 | 0.996471214 |
| CXCL2 | 1.262318534 | 7.569156682 | 0.009726 | 0.996471214 |
| HDC | 1.226030525 | 5.585964468 | 0.01884 | 0.996471214 |
| ASB16 | 1.222913596 | 8.315146714 | 0.204998 | 0.996471214 |
| TPSB2 | 1.213962304 | 7.359455867 | 0.025643 | 0.996471214 |
| LOC652102 | 1.197971828 | 9.440701549 | 0.198142 | 0.996471214 |
| HLA-DRB6 | 1.191253327 | 11.26732932 | 0.141943 | 0.996471214 |
| CD14 | 1.118550328 | 9.857966027 | 0.001632 | 0.996471214 |
| FER1L4 | 1.080232805 | 5.769039649 | 0.075818 | 0.996471214 |
| CXCL1 | 1.078805469 | 5.29133153 | 0.000307 | 0.996471214 |
| LOC647460 | 1.072982306 | 7.167243027 | 0.03703 | 0.996471214 |
| IKZF1 | 1.05646036 | 8.454827647 | 0.127499 | 0.996471214 |
| LHX6 | 1.052837399 | 5.573433897 | 0.035705 | 0.996471214 |
| LOC652113 | 1.032180298 | 6.219920828 | 0.011613 | 0.996471214 |
| XBP1 | 1.022810206 | 11.5633143 | 0.033168 | 0.996471214 |
| LOC401845 | 1.016267954 | 9.520485171 | 0.186813 | 0.996471214 |
| CLEC4M | 1.015334265 | 7.428610004 | 0.006243 | 0.996471214 |
| FOS | 1.014830996 | 6.715961331 | 0.09957 | 0.996471214 |
| IGH | 1.008697981 | 6.798522109 | 0.112178 | 0.996471214 |
| TXNDC5 | 1.008311328 | 9.03910361 | 0.050342 | 0.996471214 |
| WDR11 | -1.07949428 | 6.735337555 | 0.01003 | 0.996471214 |
| RPS26 | -1.25820049 | 11.2687166 | 0.003416 | 0.996471214 |
| MIR130A | -1.17333581 | 6.326805237 | 0.011929 | 0.996471214 |
| PRKAR1A | -1.68887593 | 10.38948961 | 0.004284 | 0.996471214 |

**Supplementary Table 7. Top differential expressed genes of microarray analyses in ACPA negative RA and PsA compared to HC**


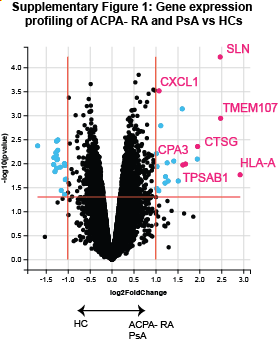


**Supplementary Figure 1 Gene expression profiling of LN biopsies from ACPA negative RA and PsA compared to healthy individuals**

Volcano plot depicting differentially expressed genes of ACPA- RA and PsA compared to HCs. Cut of values of p = 0.05 and log2-foldchange = 1.0 are represented by red lines. Healthy control (HC), anti-cyclic citrullinated protein antibodies (ACPA), rheumatoid arthritis (RA) and psoriatic arthritis (PsA)
